# Risk factors for death in tuberculosis patients requiring ICU care

**DOI:** 10.1017/S0950268820003106

**Published:** 2021-01-05

**Authors:** Jun-Li Wang, Qi-Yun Yin, Chao Han, Feng-Lin Liu, Mao-Shui Wang

**Affiliations:** 1 Department of Lab Medicine, The Affiliated Hospital of Youjiang Medical University for Nationalities, Baise, China; 2 Department of Intensive Care Unit, The First Affiliated Hospital of Shandong First Medical University, Jinan, China; 3 Department of Geriatrics, Shandong Mental Health Center, Jinan, China; 4 Department of Thoracic Surgery, Shandong Provincial Chest Hospital, Cheeloo College of Medicine, Shandong University, Jinan, China; 5 Department of Lab Medicine, Shandong Provincial Chest Hospital, Cheeloo College of Medicine, Shandong University, Jinan, China

**Keywords:** Death, hazard ratio, intensive care unit, prognostic factor, tuberculosis

## Abstract

The prognostic factor for in-hospital mortality in tuberculosis (TB) patients requiring intensive care unit (ICU) care remains unclear. Therefore, a retrospective study was conducted aiming to estimate the in-hospital mortality rate and the risk factors for mortality in a high-burden setting. All patients with culture-confirmed TB that were admitted to the ICU of the hospital between March 2012 and April 2019 were identified retrospectively. Data, such as demographic characteristics, comorbidities, laboratory measures and mortality, were obtained from medical records. The Cox proportional hazards regression model was used to identify prognostic factors that influence in-hospital mortality. A total of 82 ICU patients with confirmed TB were included in the analysis, and 22 deaths were observed during the hospital stay, 21 patients died in the ICU. In the multivariable model adjusted for sex and age, the levels of serum albumin and white blood cell (WBC) count were significantly associated with mortality in TB patients requiring ICU care (all *P* < 0.01), the hazard ratios were 0.8 (95% confidence interval (CI): 0.7–0.9) per 1 g/l and 1.1 (95% CI: 1.0–1.2) per 1 × 10^9^/l, respectively. In conclusion, in-hospital mortality remains high in TB patients requiring ICU care. Low serum albumin level and high WBC count significantly impact the risk of mortality in these TB patients in China.

## Introduction

Tuberculosis (TB) remains the first cause of death from infectious disease worldwide despite available effective therapies. In 2016, there were an estimated 10.4 million new cases of TB, 490 000 new cases with multidrug-resistant TB (MDR-TB), and 1.7 million cases died from TB [1].

Occasionally, patients with TB disease may require intensive care unit (ICU) admission, the proportion has been variably reported; it was seen in approximately 1–3% of all patients with TB [2–4]. Several studies have shown that TB patients requiring admission to the ICU carry a high mortality rate. The in-hospital mortality has been reported to be 25–63% [5–8], and the mean survival of patients who died was 53.6 days, and 50% of the patients died within the first 32 days [5]. Delayed diagnosis and/or treatment may lead to severe clinical forms of the disease and are associated with higher mortality rates [9, 10]. Many studies have evaluated risk factors for death in the treatment of TB, and many factors, such as age, sex, bacteriological status, comorbidities, immune and nutritional status of host and substance abuse, have been identified [11]. However, the prognostic factor for in-hospital mortality in ICU TB patients remains unclear.

In the current study, we aimed to document the characteristics of TB patients requiring ICU care and to estimate the in-hospital mortality rate and the risk factors for mortality in a high-burden setting.

## Methods

The retrospective study was approved by the Ethics Committee of Shandong Provincial Chest Hospital (SPCH). Written informed consent was waived because of the retrospective design of the study and the anonymous nature of the data collection.

All patients with culture-confirmed TB that were admitted to the ICU of the Shandong Provincial Chest Hospital, Jinan, China, between March 2012 and April 2019 were identified retrospectively. The hospital is a provincial public referral TB hospital, with most of patients admitted are from rural areas.

The following data were retrieved from the medical records: demographic characteristics (such as age, sex, molecular test use, TB contact history and smoking habits), comorbidities (such as forms of TB diseases, cancers and symptoms), laboratory measures (such as clinical chemistry, complete blood cell count and blood gas analysis), length of hospital/ICU stay and mortality. In addition, if multiple measurements for the same lab test were performed, the first one was collected and used for the statistical analysis.

For these analyses, death was defined as dying during treatment irrespective of cause. Time of delay was defined as the time from symptoms onset to hospital arrival. The overall survival time was defined as the interval from symptoms onset to discharge from hospital or death. Acute respiratory distress syndrome (ARDS) was diagnosed based on the criteria defined in the American-European consensus conference [12]. The outcome was defined as death and hospitalisation time, which spent in the hospital from admission to discharge from hospital or death.

Data are expressed either as mean ± s.d. (continuous variables) or as a percentage for the group (categorical variables). Categorical variables were compared using the *χ*^2^ test or Fisher's exact test, and continuous variables were compared using the Mann–Whitney *U* test or *t* test depending on the distribution of the data. Data were compared between survivors and patients who died during the hospital stay. The Cox proportional hazards regression model was used to identify prognostic factors that influence in-hospital mortality. Variables with a threshold value lower than 0.10 from univariate analysis were included in the multivariate model. Stepwise regression was used to inform the selection of a final model and *P* value less than 0.05 as a criterion to stay in the model.

## Results

### Patient characteristics

**Fig. 1.**
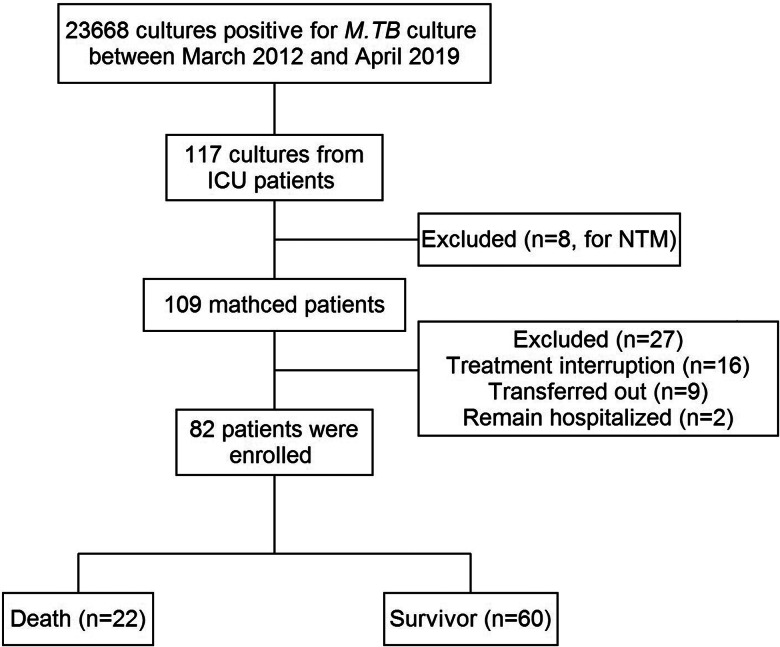
Flowchart for the selection of study patients.

Demographic, clinical and laboratory characteristics of the enrolled patients are shown in Table 1. Between March 2012 and April 2019, a total of 82 ICU patients with confirmed TB were included in the analysis, the corresponding flowchart is shown in Figure 1. A total of 22 deaths were observed during the hospital stay, 21 patients died in the ICU. Of the 82 participants, 74% were male. Age of the study participants ranged from 1 to 90 years, and the mean age was 50.6 ± 24.3 years. Eleven cases (13%) had a TB contact history. Twenty-nine patients (35%) had a smoking habit. Sixty-five patients were screened for HIV infection and all were HIV-negative. Radiographic findings for the chest were abnormal in 100% of all cases (*n* = 70) examined with chest X-ray or computerised tomography.

**Table 1.** Baseline characteristics of the patients

Characteristics	Total (*n*)	Survivals (*n*)	Deaths (*n*)	*P*	Hazard ratio (95% CI)
Demographics					
*N*	82	60 (73%)	22 (27%)		
Age (years)	51 ± 24	49 ± 25	54 ± 22	0.40	1.0 (1.0–1.0)
Sex, male	61 (74%)	47 (78%)	14 (64%)	0.34	0.6 (0.3–1.6)
Hospitalisation time (days)	51 ± 67	54 ± 55	42 ± 93		
ICU stay time (days)	30 ± 54	30 ± 54	40 ± 93	0.09	1.0 (1.0–1.0)
Time of delay (days)	35 (15, 180)	30 (14, 180)	60 (20, 180)	0.46	1.0 (1.0–1.0)
Overall survival time (days)	503 ± 1245	454 ± 1144	638 ± 1507	0.59	1.0 (1.0–1.0)
TB contact history (%)	11 (13%)	8 (13%)	3 (14%)	0.87	0.9 (0.3–3.1)
Smoker (%)	29 (35%)	22 (37%)	7 (32%)	0.94	1.0 (0.4–2.6)
Smoking habit (package-years)	9.2 ± 16.7	9.5 ± 16.7	8.2 ± 17.1	0.93	1.0 (1.0–1.0)
Clinical characteristics					
Pulmonary TB (%)	74 (90%)	55 (91.7%)	19 (86.4%)	0.70	0.8 (0.2–2.7)
Tuberculous meningitis (%)	30 (37%)	22 (36.7%)	8 (36.4%)	0.55	0.8 (0.3–1.9)
Retreatment TB (%)	16 (20%)	13 (21.7%)	3 (13.6%)	0.85	0.9 (0.3–3.1)
Disseminated TB (%)	14 (17%)	8 (13.3%)	4 (18.2%)	1.00	1.0 (0.3–3.0)
ARDS (%)	29 (35%)	15 (25.0%)	14 (63.6%)	0.004	3.7 (1.5–9.0)
Cardiovascular diseases (%)	23 (28%)	16 (26.7%)	7 (31.8%)	0.56	1.3 (0.5–3.5)
Diabetes mellitus (%)	9 (11%)	6 (10.0%)	3 (13.6%)	0.26	2.0 (0.6–7.0)
Cancers (%)	4 (5%)	2 (3.3%)	2 (9.1%)	0.100	3.5 (0.8–15.6)
Fever (%)	51 (62%)	37 (61.7%)	14 (63.6%)	0.61	0.8 (0.3–2.0)
Neurological symptoms (%)	26 (32%)	20 (33.3%)	6 (27.2%)	0.53	0.7 (0.3–1.9)
Laboratory measures					
Microbiological assays					
Acid-fast bacilli smear (+)	37 (37/77, 48%)	21 (21/56, 37.5%)	16 (16/21, 76.2%)	0.005	4.8 (1.6–14.6)
PCR (+, RT-PCR or Xpert)	44 (44/64, 69%)	32 (32/49, 65.3%)	12 (12/15, 80.0%)	0.48	1.6 (0.4–5.8)
Clinical chemistry					
Alanine aminotransferase (U/l)	44.0 ± 90.3	46.4 ± 99.9	37.5 ± 57.6	0.69	1.0 (1.0–1.0)
Aspartate aminotransferase (U/l)	61.5 ± 189.0	67.0 ± 220.5	46.5 ± 29.2	0.82	1.0 (1.0–1.0)
Total protein (g/l)	59.8 ± 9.6	61.7 ± 8.8	54.5 ± 10.0	0.002	0.9 (0.9–1.0)
Albumin (g/l)	32.1 ± 5.9	33.6 ± 5.5	27.9 ± 4.9	<0.001	0.8 (0.8–0.9)
Total bilirubin (μmol/l)	17.6 ± 13.5	18.5 ± 14.8	15.3 ± 9.0	0.31	1.0 (0.9–1.0)
Direct bilirubin (μmol/l)	9.9 ± 9.8	10.3 ± 10.8	8.8 ± 6.4	0.59	1.0 (0.9–1.0)
Urea nitrogen (mmol/l)	8.5 ± 8.3	6.8 ± 4.7	13.1 ± 13.1	<0.001	1.1 (1.0–1.1)
Creatinine (μmol/l)	79.5 ± 97.6	72.4 ± 75.0	98.6 ± 142.8	0.05	1.0 (1.0–1.0)
Creatinine kinase (U/l)	118.9 ± 172.8	134.2 ± 192.8	75.9 ± 87.2	0.29	1.0 (1.0–1.0)
Lactate dehydrogenase (U/l)	413.2 ± 464.4	386.0 ± 511.6	489.4 ± 292.3	0.49	1.0 (1.0–1.0)
*α*-Hydroxybutyrate dehydrogenase (U/l)	331.5 ± 345.3	301.0 ± 367.6	416.8 ± 262.9	0.34	1.0 (1.0–1.0)
Complete blood cell count					
WBC (10^9^/l)	10.8 ± 9.4	9.1 ± 4.5	15.2 ± 16.0	0.002	1.1 (1.0–1.2)
Red blood cells (10^12^/l)	3.85 ± 0.81	3.89 ± 0.78	3.72 ± 0.90	0.29	0.7 (0.4–1.3)
Haemoglobin (g/l)	108 ± 22	111 ± 22	102 ± 22	0.07	1.0 (1.0–1.0)
Haematocrit (%)	32.8 ± 6.1	33.3 ± 5.9	31.4 ± 6.5	0.09	0.9 (0.9–1.0)
Mean cell volume (fl)	86.1 ± 7.5	86.3 ± 6.6	85.4 ± 9.7	0.39	1.0 (0.9–1.0)
Mean corpuscular haemoglobin (pg)	28.3 ± 2.7	28.5 ± 2.7	27.7 ± 2.8	0.17	0.9 (0.7–1.1)
Mean corpuscular haemoglobin concentration (g/l)	329 ± 16	331 ± 16	325 ± 16	0.29	1.0 (1.0–1.0)
Platelets (10^9^/l)	238 ± 123	247 ± 117	211 ± 138	0.30	1.0 (1.0–1.0)
Neutrophil (10^9^/l)	9.41 ± 8.91	7.77 ± 4.04	13.86 ± 15.23	0.001	1.1 (1.0–1.2)
Lymphocyte (10^9^/l)	0.94 ± 0.76	0.97 ± 0.79	0.86 ± 0.66	0.59	0.8 (0.4–1.6)
Monocyte (10^9^/l)	0.46 ± 0.32	0.48 ± 0.30	0.38 ± 0.39	0.05	0.2 (0.0–1.0)
Eosinophil (10^9^/l)	0.05 ± 0.08	0.05 ± 0.06	0.07 ± 0.12	0.69	0.3 (0.002–64)
Basophil (10^9^/l)	0.01 ± 0.01	0.01 ± 0.01	0.01 ± 0.01	0.90	6.7 (0.0–9016.0)
Red blood cell distribution width-CV (%)	15.2 ± 2.7	14.9 ± 2.5	16.0 ± 3.1	0.16	1.1 (1.0–1.2)
Platelet distribution width-CV (%)	12.2 ± 3.4	11.9 ± 3.4	13.1 ± 3.4	0.16	1.1 (1.0–1.2)
Erythrocyte sedimentation rate (mm/h)	50.3 ± 33.3	49.3 ± 31.3	53.1 ± 39.2	0.51	1.0 (1.0–1.0)
Blood gas analysis					
pH	7.44 ± 0.09	7.45 ± 0.09	7.42 ± 0.09	0.21	0.1 (0.0–5.8)
*P*CO_2_ (mm Hg)	40.7 ± 15.1	38.5 ± 11.6	46.1 ± 20.9	0.09	1.0 (1.0–1.0)
*P*O_2_ (mm Hg)	88.8 ± 47.2	93.6 ± 52.2	77.1 ± 29.6	0.15	1.0 (1.0–1.0)
Lactate (mmol/l)	1.92 ± 1.76	1.94 ± 1.98	1.87 ± 1.12	0.87	1.0 (0.8–1.2)
Ionised calcium (mmol/l)	1.02 ± 0.20	1.01 ± 0.21	1.05 ± 0.18	0.56	2.0 (0.2–19.4)

s.d., standard deviation; HIV, human immunodeficiency virus; ICU, intensive care unit; TB, tuberculosis; ARDS, acute respiratory distress syndrome; CV, coefficient of variation.

The duration of these symptoms from onset to hospital arrival was 35 (median, interquartile range: 15, 180) days (defined as the time of delay in Table 1), the overall survival time was 503 ± 1245 days, the mean ICU stay time was 30 ± 54 days.

Of the participants, 74 patients (90%) had pulmonary TB, 30 (37%) tuberculous meningitis and 16 (20%) retreatment TB. Disseminated TB was diagnosed in 14 cases (17%). Among the 22 patients with the drug susceptibility test of *M. TB* isolates, two were confirmed as MDR-TB.

The most frequent comorbidities listed were as follows: ARDS 35%, cardiovascular diseases 28%, diabetes mellitus 11% and cancers 5%. The most common symptoms were fever (62%). In total, 32% of the patients presented with neurological symptoms (such as headache, mental confusion and coma).

The mean periods between the admission and laboratory examinations were as follows: complete blood cell count, 1.7 ± 5.6 days; arterial blood gases analysis, 4.6 ± 17.4 days and serum chemistry analysis, 1.7 ± 3.6 days. These detailed results were also presented in Table 1.

### Univariate associations with mortality

Patient characteristics and their associations with in-hospital mortality were analysed with Cox proportional hazards regression analysis (Table 2). In the study, associations with mortality were observed for ARDS (hazard ratio: 3.7, 95% confidence interval (CI): 1.5–9.0, *P* = 0.004).

**Table 2.** Cox regression analysis of mortality for demographic, clinical and laboratory characteristics

Characteristics	Multivariable	
	*P*	Hazard ratio adjusted for age and sex (95% CI)
Albumin per 1 g/l		0.8 (0.7–0.9)
WBC per 1 × 10^9^/l		1.1 (1.0–1.2)

95% CI, 95% confidence interval; ARDS, acute respiratory distress syndrome.

For laboratory measures, we also observed several variables associated with mortality, such as low levels of total protein and albumin, high levels of urea nitrogen, white blood cell (WBC) count and neutrophil count (all *P* < 0.01, Table 2).

### Multivariate associations with mortality (sex-adjusted)

Table 2 also presents two parameters that are significantly associated with mortality in the multivariable model, namely, albumin and WBC count. In the multivariable model adjusted for sex and age, the levels of albumin and WBC count were significantly associated with mortality in TB patients requiring ICU care (all *P* < 0.01), the hazard ratios were 0.8 (95% CI: 0.7–0.9) per 1 g/l and 1.1 (95% CI: 1.0–1.2) per 1 × 10^9^/l, respectively.

## Discussion

Currently, in China, few studies were performed to evaluate the in-hospital mortality of TB cases requiring ICU care and their corresponding prognostic factors. In the study, it was found that the in-hospital mortality rate in TB patients requiring ICU care was 27%. Moreover, a number of risk factors associated with mortality were identified, such as albumin and WBC count.

In the study, the mortality rate appears to be consistent with what has been reported previously in another literature [5]. However, it was below what was reported in a meta-analysis of the outcome of critically ill subjects with TB (ICU: 48% (95% CI: 41–55) [13]. Although the mortality rate reported in the study appears low when compared to the meta study, the mortality rate of 27% is high when compared to other highly lethal acute diseases [14].

The most common reasons for ICU admission of TB patients are the development of ARDS [6, 7] and severe organ failure, such as renal failure [8, 9]. In a recent study, several primary causes for ICU admission were reported in patients with TB as follows: ARDS (63%), cardiopulmonary arrest (10%), septic shock (8%), sepsis (6%) and altered sensorium (6%). However, in the study, the most common reason was tuberculous meningitis (37%), which is the most severe form of TB diseases, followed by ARDS (35%), cardiovascular diseases (28%) and disseminated TB (17%). It is worth to be noted that, although all patients were HIV-negative in the cohort, disseminated TB was frequent. As is known, HIV-infected patients have been reported to be more likely to disseminated TB [15]. Therefore, the high frequency of disseminated TB in our study may indicate a high severity of these patients.

Univariate analysis identified ARDS, low levels of total protein and albumin, and high levels of urea nitrogen, WBC and neutrophil count as being significantly associated with mortality. Multivariate analysis showed that low albumin and high WBC count were the only two significant independent predictors of death. The variables for predicting outcomes, such as serum albumin and urea nitrogen, are objective, which is easily performed, inexpensive and available in most hospital laboratories. Therefore, these important variables should be considered by physicians for evaluating the severity of illness in ICU TB patients.

Previous studies have shown that lower serum albumin was associated with infection and increased mortality in the critically ill subjects [16–18]. Hypoalbuminaemia was considered as an independent risk factor associated with mortality in community-acquired pneumonia (CAP) [19]. Similarly, Viasus *et al*. found that hypoalbuminaemia was significantly associated with the primary outcome in CAP [20]. This is not surprising because albumin plays an important role in inflammation and suggests a state of malnutrition of patients [16]. Additionally, the initial value of serum albumin might also be a result of malnutrition and underlying disease that can worsen the nutritional status of the patient. Moreover, cytokines have a negative effect on albumin expression in the liver [21]. Remarkably, in a recent meta-analysis, the administration of albumin-containing solutions for the resuscitation of patients with sepsis was associated with lower mortality compared with other fluid resuscitation regimens [22]. This implied that the administration of albumin may improve the outcome of TB patients requiring ICU care.

Elevated WBC count is a well-known indicator for severe pneumonia and served as a diagnostic biomarker in pulmonary infections [23–25]. In several studies, it was demonstrated that high WBC count was an independent predictor of in-hospital mortality of pneumonia [23, 26, 27]. Moreover, the WBC count has been reported to correlate with mortality in patients with any infection [28]. Hence, a high WBC count may relate to the severity of lung involvement in TB patients and gives an estimated risk of their mortality. In the study, we found that high WBC count was significantly associated with mortality in TB patients requiring ICU care. In a previous study by Kim *et al*., a similar result was reported that an increased value of WBCs was significantly associated with mortality in TB patients.

Our study also has some limitations. First, the sample size is relatively small, although the study included all consecutive patients who had ICU care in a 7-year period, this may limit the generalisability of the findings. Second, all information was retrieved retrospectively from medical records and probably was not uniformly complete and accurate. In fact, due to data availability, several important factors, such as C-reactive protein and procalcitonin which have been recognised as risk factors for the mortality in TB patients and may improve the management of TB patients requiring ICU stay [29–32], were not included for analysis. Third, the study has a long duration. This may have significant effect on treatment regimens, criteria for ICU admission and others. Fourth, unfortunately, several factors were significant in the univariate analysis, which were also reported in other studies, such as sex, ARDS, cancer and haemoglobin [11, 14]. However, when added to multivariate models, none of these variables reached significance. This may be due to the small sample size, confounding effect or weak associations. We believe that a larger prospective study, which has sufficient statistical power would be helpful to understand the baseline characteristics associated with mortality.

## Conclusions

In conclusion, our study found a relatively high in-hospital mortality rate in TB patients requiring ICU care. Low albumin level and high WBC count could serve as prognostic factors for death in these TB patients in China. These findings are important for the appropriate management of these patients and may improve the outcome. Nevertheless, additional studies are necessary to confirm these data, taking into account other possible risk factors in the univariate analysis.

## Data Availability

The data that support the findings of this study are available from the corresponding author (WMS) upon a reasonable request.
